# Insights into metabolic disease from studying genetics in isolated populations: stories from Greece to Greenland

**DOI:** 10.1007/s00125-016-3926-3

**Published:** 2016-03-18

**Authors:** Eleftheria Zeggini, Anna L. Gloyn, Torben Hansen

**Affiliations:** Wellcome Trust Sanger Institute, Wellcome Trust Genome Campus, Hinxton, Cambridge, CB10 1HH UK; Oxford Centre for Diabetes Endocrinology & Metabolism, University of Oxford, Oxford, UK; Wellcome Trust Centre for Human Genetics, University of Oxford, Oxford, UK; Oxford NIHR Biomedical Research Centre, Churchill Hospital, Oxford, UK; The Novo Nordisk Foundation Center for Basic Metabolic Research, Faculty of Health and Medical Sciences, University of Copenhagen, Copenhagen, Denmark; Faculty of Health Sciences, University of Southern Denmark, Odense, Denmark

**Keywords:** Genetic variants, Genome-wide association studies, Isolated populations, Low-frequency variants, Next-generation sequencing technology, Review, Type 2 diabetes

## Abstract

Over the last 10 years substantial progress has been made in our understanding of the genetic basis for type 2 diabetes and related traits. These developments have been facilitated by technological advancements that have allowed comprehensive genome-wide assessments of the impact of common genetic variation on disease risk. Current efforts are now focused on extending this to genetic variants in the rare and low-frequency spectrum by capitalising on next-generation sequencing technologies. This review discusses the important contributions that studies in isolated populations are making to this effort for diabetes and metabolic disease, drawing on specific examples from populations in Greece and Greenland. This review summarises a presentation given at the ‘Exciting news in genetics of diabetes’ symposium at the 2015 annual meeting of the EASD, with topics presented by Eleftheria Zeggini and Torben Hansen, and an overview by the Session Chair, Anna Gloyn.

The search for low-frequency and rare variants associated with complex traits requires very large sample sizes to attain the necessary power to detect these effects. Population isolates offer the opportunity to discover such signals with much lower sample sizes. Isolated, or founder, populations have typically gone through a bottleneck event, followed by population expansion with limited rates of migration. These characteristics can offer power advantages in the study of complex trait genetics, especially with respect to the identification of association signals at low frequency, and rare variants that would otherwise be difficult or impossible to detect in cosmopolitan populations [1, 2]. Some rare variation present in the source population may have been lost in the isolated population, and this can reduce the number of neutral rare variants, which in turn can increase the power of burden tests (i.e. association tests looking for an aggregation of multiple rare variants in a functional unit, e.g. gene or regulatory region). Other rare variants that have made it through the bottleneck can increase in frequency due to drift, or in some cases, selection. This in turn increases the power of association studies as smaller sample sizes are required to detect individual risk loci for complex diseases and traits. In addition, the genomes of isolates have been shaped by population history to typically have longer stretches of linkage disequilibrium. Founder populations also tend to have more homogeneous environmental exposures and, in some cases, deep information on genealogy. These attributes can be further enhanced through the ability to recall participants for further characterisation based on genotype and through access to their medical records. Over the last few years, the field of complex trait genetics has witnessed a flourishing of genetic association studies focusing on isolated populations, with the majority of findings replicating across cosmopolitan populations [3–12]. Here, we outline examples from two founder populations stemming from geographical coordinates in Europe ranging from the extreme North and South: Greek individuals from the island of Crete in the south Mediterranean and Inuit individuals from the island of Greenland in the Arctic (Fig. 1).

**Fig. 1:**
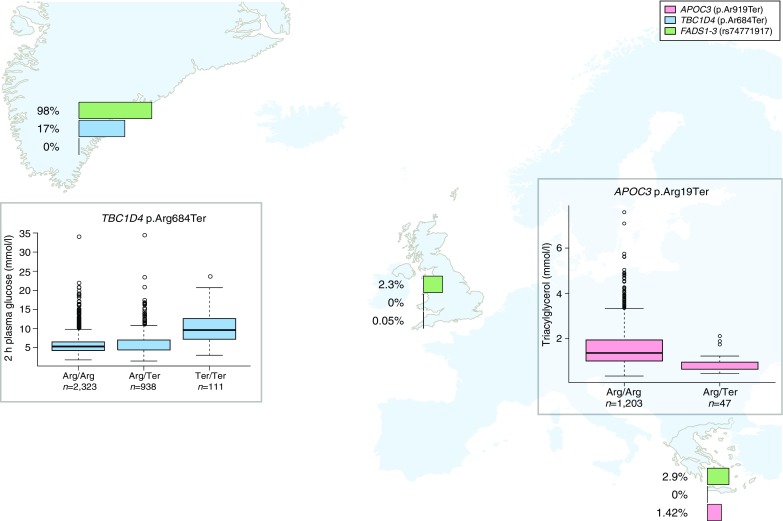
Allele frequencies (displayed on log scale) of isolate-enriched variants in Greenland, Crete and the UK general population (UK10K frequencies are taken from www.uk10k.org [21]). Frequency bars are pink for *APOC3* p.Arg19Ter (rs76353203) [11], blue for *TBC1D4* p.Arg684Ter (rs61736969) [7] and green for *FADS2* rs7477191 [18]. The larger graphs show box plots of blood triglyceride levels by *APOC3* p.Arg19Ter genotype in Crete, and plasma glucose levels by *TBC1D4* p.Arg684Ter in Greenland

The inhabitants of the island of Crete in Greece have in the past been investigated through epidemiological studies focusing on dietary and nutrition patterns with respect to metabolic disease [13, 14]. The Hellenic Isolated Cohorts (HELIC) Minoan Isolates (MANOLIS) study was launched in 2008 with the explicit aim of studying the genetic basis of metabolically relevant complex traits in the isolated population residing in the villages of the mountainous Mylopotamos region of Crete (www.helic.org). Anecdotally, this population is known to enjoy good health in old age despite a diet high in animal fat. A total of 1600 recontactable individuals (making up >10% of the total population) were recruited into the study and subjected to extensive phenotyping, including anthropometry, detailed diet and lifestyle information, medical history, and blood sample collection for DNA extraction as well as biochemical, haematological and other biomarker analyses. Following genome-wide genotyping and low-depth whole genome sequencing of the entire cohort, association analysis was carried out for quantitative traits of metabolic relevance measured in the population (such as fasting glucose and insulin levels, lipid traits and anthropometry).

Initial population genetic analyses revealed statistically significant enrichment of missense (and therefore potentially functional coding) variants that have substantially risen in frequency in the isolate compared with the general Greek population [15]. A genome-wide scan for association with lipid traits identified a cardioprotective effect at a nonsense variant, p.Arg19Ter, in the *APOC3* gene. The variant abrogates expression of all three isoforms of the gene and has a 40-fold higher frequency in MANOLIS compared with the source population (Fig. 1) [11]. The same variant had previously been found to have risen to similar frequency and to have a similar protective association with lipid levels in the Old Order Amish, an independent founder population [8]. Haplotype analysis demonstrated that the variant predates the divergence of both isolates from the general European population. Interestingly, the Amish also have a diet high in animal fat, indicating that the variant may have risen in frequency because of its cardioprotective effect; however, further analyses were not powered to detect selection signatures due to the low allele frequency (~2%) [11]. No homozygous individuals were observed in either founder population. Recent work has shown that oligonucleotide-mediated inhibition of *APOC3* has therapeutic potential as it decreases blood triacylglycerol (triglyceride) levels [16]. Further recent studies have also identified associations between a burden of multiple loss-of-function (LoF) variants in the *APOC3* gene and lipid levels in the general European population [5, 17]. This story represents a proof-of-principle for the power of isolated populations in medically relevant trait locus discovery, exemplified by an early finding with significant translational potential that is transferable to cosmopolitan populations.

The Greenlandic population is a historically small and isolated founder population. It stands out in at least one important way in comparison with other well-studied founder populations, such as the Finnish and Icelandic populations: these other founder populations are all genetically similar to at least one large population, whereas the Greenlandic Inuit are not closely related to any large population [18]. The Greenlandic Inuit and indigenous populations throughout the Arctic have different fat distribution compared with Europeans [19]. Furthermore, the isolated Greenlandic Inuit population has lower levels of plasma glucose and serum insulin and higher levels of HDL cholesterol than Danes at any given level of obesity [19]. Little is known about how genetics and lifestyle behaviour, particularly physical activity, interact to influence metabolic health in Inuit and whether a recent risk increase of developing type 2 diabetes and related cardiometabolic conditions is driven by a transition from a traditional subsistence lifestyle to a modern, industrialised way of life.

A common Greenlandic Inuit-specific nonsense p.Arg684Ter variant (in which arginine is replaced by a termination codon) in the gene *TBC1D4*, with an allele frequency of 17%, was recently discovered (Fig. 1). It was shown that homozygous carriers of this variant have markedly elevated 2 h serum insulin levels, postprandial hyperglycaemia, impaired glucose tolerance and a tenfold increased risk of type 2 diabetes [7]. The variant explains about 15% of type 2 diabetes occurrence in Greenland. Analyses of skeletal muscle biopsies in groups of three individuals carrying zero, one or two copies of p.Arg684Ter revealed that hetero- and homozygous carriers of this stop-gained variant in the gene coding for TBC1D4 are characterised by a marked decrease in the RNA and protein expression of the skeletal muscle-specific long isoform of TBC1D4. Other tissues such as pancreatic beta cells, adipose tissue and liver tissue expressing the short isoform of TBC1D4 were not affected by the stop-gained mutation [7]. Thus, homozygous carriers of p.Arg684Ter could be considered as human muscle-specific knockouts of *TBC1D4*. Since the variant is common in Greenland (allele frequency of 17%) and rare/absent in all other populations, it is possible that selection has historically favoured it in Inuit, possibly because of the low carbohydrate diet associated with traditional Inuit lifestyle. Indeed, weak evidence for positive selection was found [7]. The finding of a common tissue-specific and Inuit-specific LoF variant constitutes proof-of-concept that conducting genetic association studies in small and isolated populations can reveal population-specific type 2 diabetes risk variants and novel biological insights.

While other LoF variants in Inuit may have increased in frequency by chance (genetic drift), it is conceivable that some variants are common specifically in Inuit because of adaptation to the special climate and environment in the Arctic. In fact, a recent study of signs of selection identified genetic variants in fat metabolism in the region of the *FADS1-3* genes that alter metabolism of the large amounts of polyunsaturated fatty acids found in the traditional Inuit seafood diet. Gene variants under selection have a strong effect on height and weight, of up to 2 cm and 4 kg, respectively, as well as a protective effect on cholesterol and triacylglycerol levels [20]. This study illustrates the utility of evolutionary studies of locally adapted populations for understanding the genetic basis of phenotypic variation among humans.

What is becoming increasingly clear is that rare variants of large effect are most likely to be specific to or enriched in particular ancestries. These illustrations from Greece and Greenland provide elegant examples of the opportunities for translational biology offered through the discovery of rare coding alleles that alter disease risk. Detailed physiological studies in carriers of these alleles offer the potential to understand the impact of perturbing particular biological pathways in humans, offering the chance to understand the interplay of proteins in multiple regulatory pathways shedding light on likely adverse on-target effects of therapeutic manipulation.

## Abbreviation

LoF: Loss-of-function
